# Human senses and sensors from Aristotle to the present

**DOI:** 10.3389/fneur.2024.1404720

**Published:** 2024-07-03

**Authors:** Thomas Brandt, Marianne Dieterich, Doreen Huppert

**Affiliations:** ^1^German Center for Vertigo and Balance Disorders, University Hospital, Ludwig-Maximilians-Universität Munich, Munich, Germany; ^2^Department of Neurology, University Hospital, Ludwig-Maximilians-Universität Munich, Munich, Germany

**Keywords:** Aristotle, sensory systems, sensory receptors, vestibular system, proprioception, history of perception

## Abstract

This historical review on the semantic evolution of human senses and sensors revealed that Aristotle’s list of the five senses sight, hearing, touch, taste, and smell is still in use among non-scientific lay persons. It is no surprise that his classification in the work “De Anima” (On the Soul) from 350 BC confuses the sensor “touch” with the now more comprehensively defined somatosensory system and that senses are missing such as the later discovered vestibular system and the musculotendinous proprioception of the position of parts of the body in space. However, it is surprising that in the three most influential ancient cultures, Egypt, Greece, and China—which shaped the history of civilization—the concept prevailed that the heart rather than the brain processes perception, cognition, and emotions. This “cardiocentric view” can be traced back to the “Doctrine of Aristotle,” the “Book of the Dead” in ancient Egypt, and the traditional Chinese medicine of correspondence documented in the book “Huang di Neijing.” In Greek antiquity the philosophers Empedocles, Democritus and Aristotle were proponents of the allocation of the spirit and the soul to the heart connected to the body via the blood vessels. Opponents were the pre-Socratic mathematician Pythagoras, the philosopher Plato, and especially the Greek physician Hippocrates who regarded the brain as the most powerful organ in humans in his work “De Morbo Sacro.” The Greek physician Galen of Pergamon further elaborated on the concept of the brain (“cephalocentric hypothesis”) connected to the body by a network of nerves. The fundamental concepts for understanding functions and disorders of the vestibular system, the perception of self-motion, verticality and balance control were laid by a remarkable group of 19th century scientists including Purkynӗ, Mach, Breuer, Helmholtz, and Crum-Brown. It was also in the 19th century that Bell described a new sense of a reciprocal sensorimotor loop between the brain and the muscles which he called “muscular sense,” later termed “kinaesthesia” by Bastian and defined in 1906 as “proprioception” by Sherrington as “the perception of joint and body movements as well as position of the body or body segments, in space.” Both, the vestibular system and proprioception could be acknowledged as senses six or seven. However, we hesitate to recommend “pain”—which is variously assigned to the somatosensory system or extero-, intero-, visceroception—as a separate sensory system. Pain sensors are often not specific but have multisensory functions. Because of this inconsistent, partly contradictory classification even by experts in the current literature on senses and sensors we consider it justified to recommend a comprehensive reorganization of classification features according to the present state of knowledge with an expansion of the number of senses. Such a project has also to include the frequent task-dependent multisensory interactions for perceptual and sensorimotor achievements, and higher functions or disorders of the visual and vestibular systems as soon as cognition or emotions come into play. This requires a cooperation of sensory physiologists, neuroscientists and experienced physicians involved in the management of patients with sensory and multisensory disorders.

## 1 Introduction

The historical debate on the number of human sensory systems—five, six, or seven—is still alive. It was Aristotle (384–322 BC), a Greek universal scholar, who first listed five senses “sight, hearing, touch, taste, and smell” in his seminal work “De Anima” (1). A sixth sense was proposed by the Scottish physiologist and anatomist Charles Bell (1774–1842) as the “muscular sense,” which was later termed “proprioception” by the English neurophysiologist Charles Scott Sherrington (1857–1952). Proprioception means awareness of the position or movement of the body or parts of the body relative to each other (2). Both concepts did not yet refer to vestibular labyrinthine function, which was just discovered around the same time in the 19th century. Current classifications by physiologists and physicians are often inconsistent as to the distinguishing features between sensory systems and their associated groups of sensors which together form the unity of a modality. Some examples of controversies pertain to the categorization, e.g., of touch, of proprioception, and of pain. Our review will therefore focus on different criteria of classification, in particular on definitions and delimitations of senses and sensors and on the question of whether, for example proprioception is a system entity which includes the vestibular system and whether pain is a distinct and separate sensory system or just a common multisensory perceptual quality of various exteroceptive and interoceptive sensors. This will be discussed further after a historical medical and etymological discourse about this topic over the last millennia in the most influential ancient cultures: the Greek, Chinese, and Egyptian cultures. Thus, this selective historical review does not include contributions from Greco-Persian, Roman, Arab, and Indian scientists and philosophers who referred later to these cultures, for example Avicenna (980–1,037), a Persian physician.

## 2 Historical review

### 2.1 Greek views from the pre-Socratics to Aristotle

#### 2.1.1 Pre-Socratics

In Greek pre-Socratic philosophy, there is no collective depiction of the different senses; instead, they are individually mentioned in connection with respective or other non-adequate organs. Alkmaion of Kroton (ca. 570–500 B.C.), a physician and natural scientist and student of Pythagoras, saw the eye connected to the brain as an organ of thought through special ducts (πóροι) on the basis of anatomical investigations (3, 4). Empedocles (ca. 482–420 BC), a Greek natural philosopher, used παγάμαι, meaning “palm” or ““claw” as a general term for sensory experience. The senses were understood as those that “[..] grasp their objects as if with hands.” He described the sensory activity with ἀϑρείν, “see, behold, stare,” but also “hold, hold on, support.” Both terms can be interpreted as senses of sight and touch. Democritus (460–370 BC), a presocratic and the main representative of the “atomic theory,” was the first to distinguish between perception and cognition. “Atomic theory” can be understood as the idea that the things surrounding us can be recognized through the openings of our body—the sensory organs—. The soul located inside (i.e., an atomic aggregate) or the mind (νοῦς) perceives the perceptual impressions transmitted by the senses, sometimes slightly changed, and thereby discloses the atomic structure of the environment. He assumed that human beings are mixed of elements and perceive things according to earlier perceptual experiences (5). There is fire inside the eye, which is sent out as a ray of vision onto the outside world, and this is how vision arises. Conversely, fine imprints flow off the surface of the outside world; if these signals fit in the pores of the sense organs, they are perceived (5). For Democritus, each perception is through touch. The autonomy of the “images” that invade the eye makes perception an enduring process (πάϑος). Even before Plato, he emphasized the relativity of sensory perceptions due to their lack of objectivity and separated “dark” knowledge, conveyed through the senses, from “real” knowledge, based on the mind (5). This already came close to the later work by Purkinje in the 19th century on the subjectivism of vision “The Dawning of Neuroscience” (6).

#### 2.1.2 Plato

According to Plato (427–347 BC), the mind or soul processes the sensory impressions, thus separating the sensory process from the thought process. In his work Timaios (Τίμαιος), various scientific topics are discussed in the form of a fictional dialog (e.g., between Socrates and Timaios of Lokroi), sensory perceptions (vision, hearing, smell, color, and pain) are dealt with separately. Vision worked as follows: “…Since each body in its entirety, because of the similar composition, receives the same [sensory] impressions, it transmits the movements of everything it encounters and what encounters the body up to the soul and in this way generates the perception we name seeing…” (7). The function of the crossing of the tractus opticus was also known, i.e., the inverted perception of the visual field: “but the left appears on the right because the opposite parts of the visual ray come into contact with the opposite parts [of the object’s rays]….” (7). Colors were separated from vision as a “fourth form of perception” (τέταρτον […] γένος ἡνῖν αἰσϑητικόν), based on particles of different sizes that radiate from bodies and hit certain particle sizes of the sense of sight: if the particle sizes are the same, the object is transparent, if they are different, changes in the ray of sight occur (pulling together or pushing apart), causing white or black perception. Colors originate through the penetration of particles with a stronger drive, which trigger physical changes, i.e., mixing of the elements; depending on the mixing ratio, colors such as red, yellow, gray, light brown and purple red, are created. The perception of hearing was explained as follows: “…In general, let us now take the tone as an impulse, which from the air is conveyed through the ears, brain and blood to the soul; but the resulting movement, which, beginning in the head, ends at the liver site, is called hearing. Everything that is fast in this movement creates a high-pitched sound, everything that is slower creates a deeper sound; a uniform movement causes an even and smooth sound, the opposite a rough; the violent movement causes a loud sound, each opposite movement a silent…” (7). No specific organ was assigned to the sense of touch. Pleasure and pain, as mutually antagonistic sensations, were considered to be caused by violent impulses against nature (pain) or the restoration to the natural state (pleasure). Taste was explained anatomically by sensations arising from connections and disconnections mediated by veins or tubes of the tongue, which extended to the heart and contracted with the intrusion of particles of earth of various types (rough, smooth, etc.), drying out, expanding or loosening. This was believed to create the different taste sensations such as sour, tart, bitter, pungent and sweet. Smell was only differentiated as pleasant and unpleasant.

#### 2.1.3 Aristotle

Aristotle (384–322 BC) expanded the epistemology of the pre-Socratics as well as the sensory physiology of his predecessors, theories that survived well beyond the Middle Ages and even influenced Jewish and Arabic natural philosophy. His sensory theory separates sensory perception from other processes that take place in the psyche, and does not attribute perception to direct contact between the object and the sensory organ, but assumes a mediator, the central organ (τὸ ἡγεμονικόν). The act of perception was believed to take place in the external organs themselves, in the eye, in the ear, in the external organ of smell, independently of the central sense.

“With each individual perception, one must first speak of what is perceptible in each case. Perceptible has three meanings. Two kinds of the perceptible, shall we say, are perceived in themselves, one merely accidentally. Of the two, one is specific for the particular sense, the other common to all. By peculiar I mean that which cannot be perceived by any other sense and which cannot deceive the individual, such as seeing color, hearing sound, and tasting juice; but the sense of touch is more complex. So, every sense perceives differences in them and is not mistaken as to whether there is color or noise, but only as to what and where the colored thing is or what and where the sound is. This, then, is called what is peculiar to the individual senses. What they have in common is movement, standstill, number, shape and size.”

*“Λεκτέον δὲ καϑ᾽ ἑκάστην αἴσϑησιν περὶ τῶν αἰσϑητῶν πρῶτον. Λέγεται δὲ τὸ αἰσϑητὸν τριχῶς, ὧν δύο μὲν καϑ᾽ αὑτά φαμεν αἰσϑάνεσϑαι, τὸ δὲ ἓν κατὰ συμβεβηκός. Τῶν δὲ δυοῖν τὸ μὲν ἴδιόν ἐστιν ἑκάστης αἰσϑήσεως, τὸ δὲ κοινὸν πασῶν. Λέγω δ᾽ ἴδιον μὲν ὃ μὴ ἐνδέχεται ἑτέρᾳ αἰςϑήσει αἰσϑάνεσϑαι, καὶ περὶ ὃ μὴ ἐνδέχεται ἀπατηθῆναι, οἷον ὄψις χρώματος καὶ ἀκοὴ ψόφου καὶ γεῦσις χυμοῦ, ἡ δ᾽ ἁφὴ πλείους [μὲν] ἔχει διαφοράς, ἀλλ᾽ ἑκάστη γε κρίνει περὶ τούτων, καὶ οὐκ ἀπατᾶται ὅτι χρῶμα οὐδ᾽ ὅτι ψόφος, ἀλλὰ τί τὸ κεχρωσμένον ἢ ποῦ, ἢ τί τὸ ψοφοῦν ἢ ποῦ. τὰ μὲν οὖν τοιαῦτα λέγεται ἲδια ἑκάστης, κοινὰ δὲ κίνησις, ἠρεμία, ἀριθμός, σχῆμα, μέγεϑος”* (8).

Further, he hypothesized that the sense of taste is a kind of touch, not dependent on an external medium. The medium of the sense of touch is the flesh, which coincides with all other senses in the central organ.

“Every sense-perception, then, relates to an underlying perceptual object; it takes place in the sense-organ, insofar as it is a sense-organ, and judges the differences of the underlying sense-object, such as seeing white and black, or tasting sweet and bitter; the same applies to the other perceptions.”

*“Ἑκάστη μὲν οὖν αἴσθησις τοῦ ὑποκειμένου αἰσθητοῦ ἐστίν, ὑπάρχουσα ἐν τῷ αἰσϑητηρίῳ ᾗ αἰϕϑητήριον, καὶ κρίνει τὰς τοῦ ὑποκειμένου αἰσϑητοῦ διφοράς, οἷον λευκὸν μὲν καὶ μέλαν ὅψις, γλυκὺ δὲ καὶ πικρὸν γεῦσις• ὁμοίοως δ᾿ ἔχει τοῦτο καὶ ἐπὶ τῶν ἄλλων”* (8).

Accordingly, the various external organs therefore perceive the sensory qualities to be perceived in purity, and each sense only perceives a specific kind of quality, the sense of sight the kind of color, the sense of hearing that of sound. The central sense was the principle that perceived everything (τὸ αἰσϑητικὸν πάντων), in which the act of perception took place. The organ of the central sense was the heart. The external organs could not function independently of the central sense. The central organ was the all-ruling organ with which the soul perceived everything, in which all formed just one organ. The central sense also had the function of perceiving the combination of the qualities perceived by different senses in one object or of uniting the different perceptions into the perception of an object (1). We think that this described multisensory convergence and interaction as a perceptual principle.

#### 2.1.4 Stoics

The concept of pneuma as the carrier of organic warmth, i.e., the soul, which was implanted in all living beings from their origin in the womb, used by Aristotle (9), was explained in more detail by the Stoics. Each of the five senses had its own pneuma, which they associated with the five elements. The pneuma of the smell was in the nose, described as moist and vaporous. According to the Stoics, the senses were not afferent, as in Empedocles, but efferent. The starting point of perception was the leading central organ, between which and the respective sense the pneumatic currents flowed back and forth. This theory was supported by the discovery of nerves by the Alexandrian physician Herophilus (ca. 300 BC), which were credited with transmitting the pneuma (10, 11).

### 2.2 Cardiocentric vs. Cephalocentric hypotheses in ancient Egypt/Mesopotamia, Greece, and China

#### 2.2.1 Egypt/Mesopotamia

**Figure 1:**
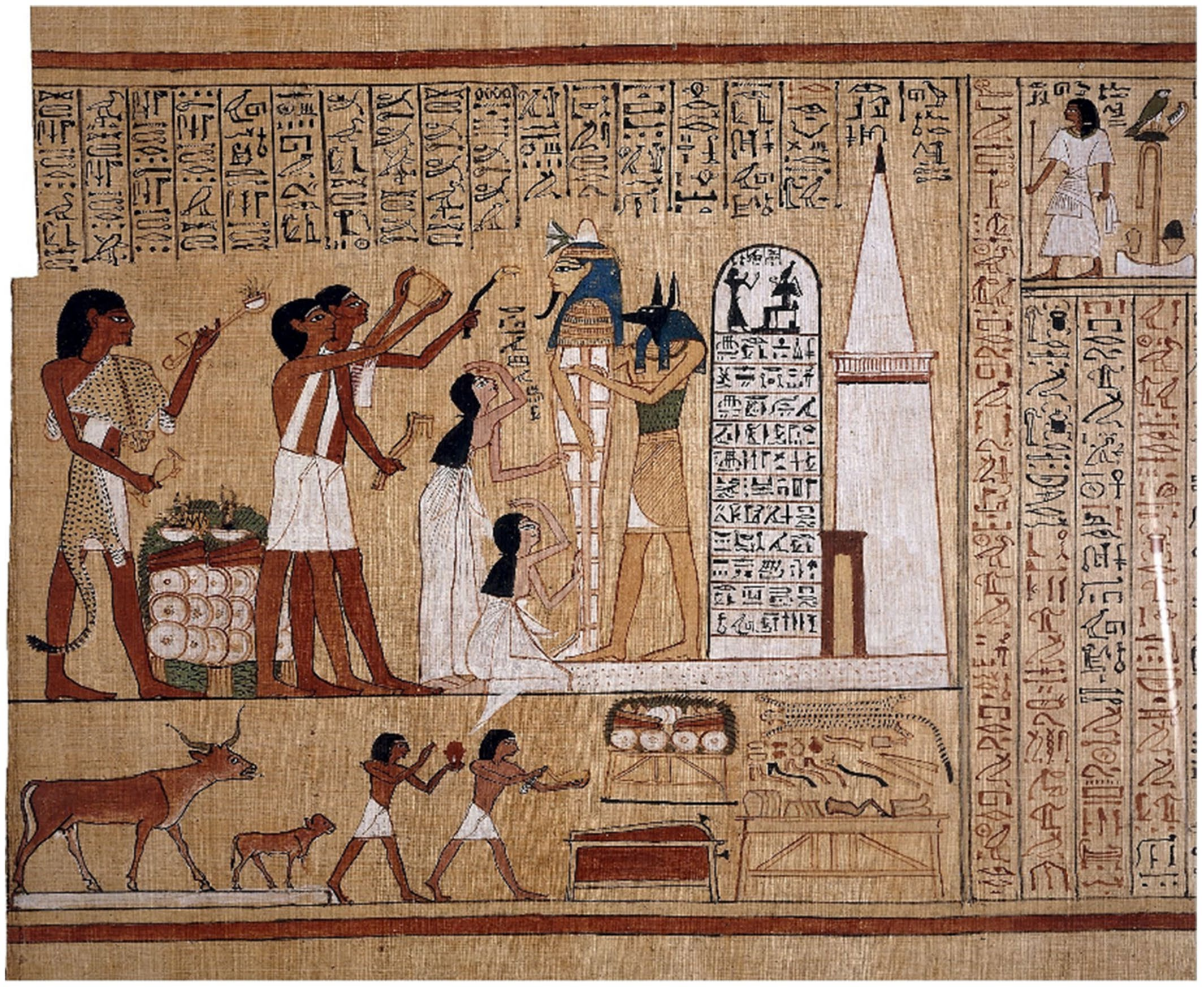
The picture shows the mouth-opening ritual from the “Book of the Dead” of Hunnefer (Papyrus Hunnefer, 1,275 BC; Collection British Museum). In the center is the mummy of Hunnefer, held to the right by the god Anubis (or a priest wearing a jackal mask). The two priests on the left with white sashes perform the ritual of opening the mouth. Tools are used to touch the mouth of the deceased so that they can speak, eat and drink again. Sacrificial sayings accompanied the ritual, embalming and sacrificial scenes (bottom left sacrifice of the severed front leg of a calf). On the right side of the lower scene is a table containing the various paraphernalia needed for the mouth opening ritual.

The cardiocentric view that the heart rather than the brain is the seat of the soul and the center of emotions, cognition, and sensorimotor control can be traced back to the “Book of the Dead*”* in ancient Egypt, the doctrine of the Greek philosopher Aristotle and traditional Chinese medicine (12, 13). The Egyptians believed that the heart was the most valuable organ and the key to a successful journey through the afterlife, which also determined their performance of mumification. This included removing the brain through the nostrils, but leaving the heart as the only organ in the corpse. The Book of the Dead consisted of individual papyrus rolls and contained hieroglyphic spells with colored illustrations such as The Mouth Opening Ritual (Figure *1*). This ritual consisted of individual scenes that were performed first on statues in the Old Kingdom. It can also be seen as a sacrificial ritual, perhaps also as an embalming ritual. Probably from the Thinite period onwards (ca. 3,100 BC), it was transferred piecemeal into a ritual for the dead on mummies, which was used both in the pyramids and in private tombs in the Old and Middle Kingdoms (14). Since the New Kingdom, the ritual has been reproduced in detail in pictures and texts (Figure 1). With tools, the mouth of the deceased was touched by a Sem-priest who assumed the god-worldly role of Horus (15). The use of the mouth was thus supposed to be ensured in the afterlife. In the Osirian belief in the dead, the dead person says to Osiris, the god of the dead: “Hail, Osiris, Behold, I am come. I am Horus, who opens thy mouth together with Ptah, who transfigures thee together with Thoth, who gives thee thy heart within, so that thou rememberest what thou hast forgotten, who makes thee eat bread at thy pleasure, more than what was done to thee on earth.” (14). Transfiguration and opening of the mouth are thus to be accomplished in the afterlife, the example of Osiris is involved, the goal lies in participation in the heavenly sacrifice (14). The dead should therefore be able to speak, eat and drink again in the hereafter. The raising of the mummy symbolizes revival (15). Other sense organs such as the eyes, nose and ears were also touched in order to regain the senses after death, i.e., to be able to see, smell and hear again. Likewise, the use of the limbs should also be possible again in the afterlife. Sacrificial sayings accompanied the ritual, incense was included in the ritual procedure (14). The removal of the brain, but preservation of the heart clearly supports the view that the ancient Egyptians believed the heart to be the organ which evaluates sensory input and sensorimotor control and was the seat of the soul.

#### 2.2.2 Greece

**Figure 2:**
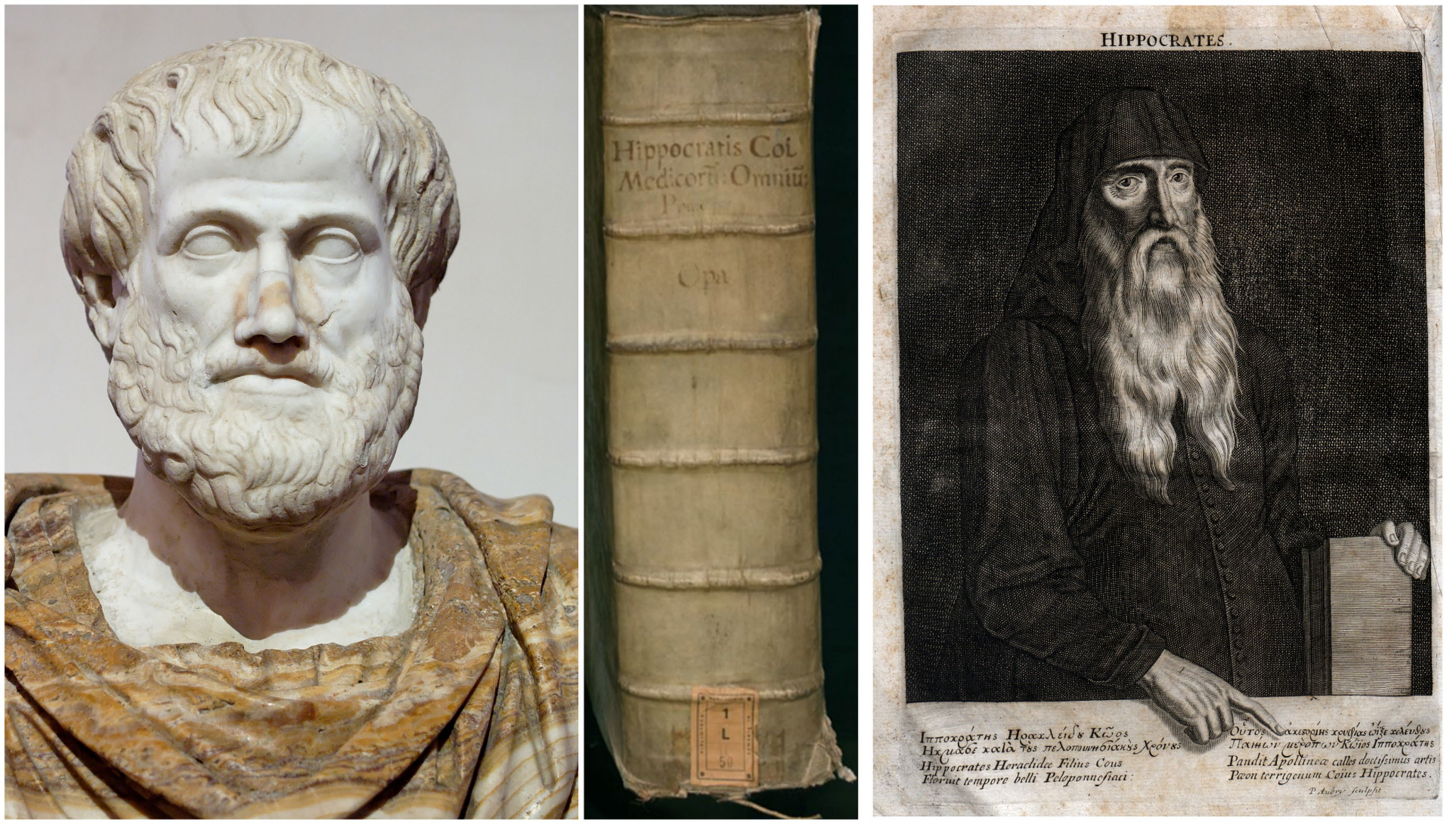
Left: Marble bust of Aristotle, Roman copy after the Greek bronze original by Lysippos (ca. 330 BC). The alabaster cloak is a modern addition (Museo Nazionale Romano di Palazzo Altemps, Rome). Right: Hippocrates of Kos. Magni Hippocratis medicorum omnium facile principis, opera omnia quae extant, 1,657 (middle). Available in the BEIC digital library and uploaded in partnership with BEIC Foundation.

Also in Greek antiquity, body and soul—especially the question of which organ accommodates the soul—were the subject of intense philosophical debate (12). The philosophers Empedocles (492–432 BC), Democritus (460–370 BC) known for his atomic theory of the universe and as one of the teachers of Hippocrates (460–370 BC) as well as the famous Aristotle were proponents of the allocation of the spirit and the soul to the heart. According to Aristotle thinking took place in the heart which was connected with all parts of the body via the blood vessels (Figure 2). Opponents of the cardiocentric hypothesis were the pre-Socratic mathematician and philosopher Pythagoras (580–489 BC) who contributed to the writing of the corpus Hippocraticum, the Greek philosopher Plato (427–347 BC) who located thinking in the brain rather the heart, and the Greek physician Hippocrates regarded as the father of medicine including the Hippocratic Oath. He taught that diseases are caused by physical pathomechanisms, not as a consequence of an annoyance of gods. In his work “De Morbo Sacro” Hippocrates wrote that the brain is a most powerful organ in humans (*“…κατὰ ταῦτα νομίζω τὸν ἐγκέφαλον δύναμιν ἔχειν πλείστην ἐν τῷ ἀνθρώπῳ ….διὸ φημὶ τὸν ἐγκέφαλον εἶναι τὸν ἑρμηνεύοντα τὴν ξύνεσιν.”*) and that those who claim that we think with the heart are mistaken (*“…λέγουσι δέ τινες ὡς καὶ φρονέομεν τῇ καρδίῃ καὶ τὸ ἀνιώμενον τοῦτ᾿ ἐστὶ καὶ τὸ φροντίζον…”*) (16) (Figure 2). The Greek physician Galen of Pergamon (~ 130–200 AD) who performed human autopsies, further elaborated on the concept that the brain is the seat of the soul connected to the body by a network of nerves (17).

#### 2.2.3 China

In ancient Chinese medicine it was also the heart which determined the understanding of sensory perceptions (18). The fundamental text in Chinese medicine of correspondences, the “Huangdi Neijing”—which was continuously revised between the 2nd century BC and the 2nd century AD—assigned the Ying organs liver, heart, spleen, lungs, and kidneys specific functions, while the brain was largely neglected or, if mentioned at all, was described as storage for a substance called “marrow.” The brain belonged to neither the Yin nor the Yang organs. The heart controlled and interpreted perception, mediated by reciprocal connections with the sensory organs (12). The ability to see clearly, for example, was based on the cardial condition of balanced emotions. The Confucian legalistic philosopher Xunzi (3rd century BC) wrote about these interconnections: “The heart possesses an overall understanding. Because of this overall understanding, it may rely upon the perception of the ear and understand sounds correctly or rely upon the perception of the eye and understand forms correctly” (19). The eyes were the recipients of the essence of various organs in the body. In addition, they stored two different forms of Qi (energy of life), the breath soul and the body soul, and let the mind Qi emerge. “When the mind (shen) is exhausted, the breath and body soul, and the will (zhi) and thoughts (yi) disperse into chaos (luan)” (19).

### 2.3 Late discovery of the vestibular sense

**Figure 3:**
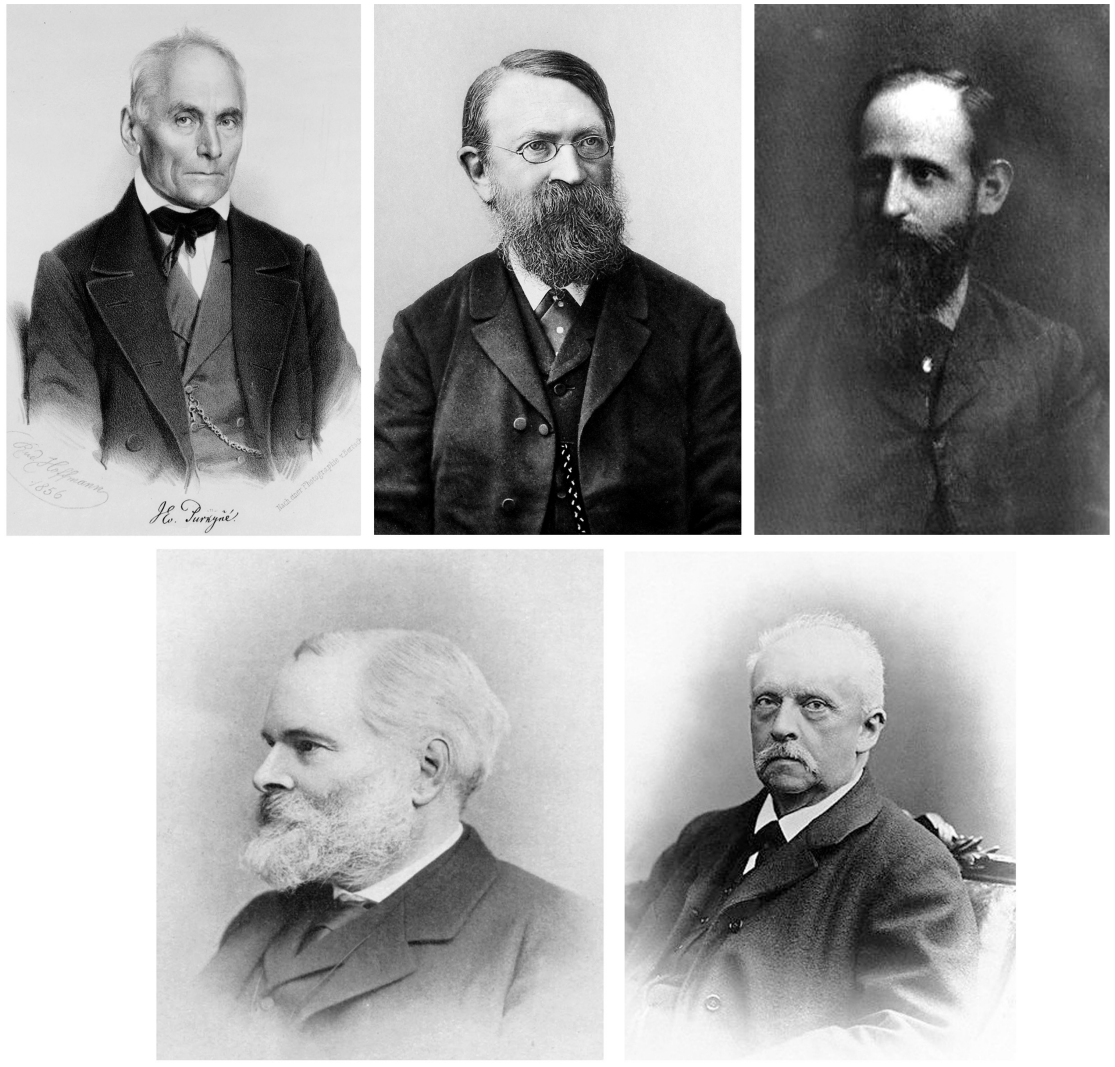
Upper row left: Jan Evangelista Purkynӗ, Czech anatomist (date 1856; available from the United States Congress’s Prints and Photographs division; https://www.loc.gov/rr/print under the digital ID cph.3c33404). Middle: Ernst Mach, Austrian physicist (date *ca.* 1903; Zeitschrift für Physikalische Chemie, Band 40, 1902). Right: Josef Breuer, Austrian physician (date 1877; Albrecht Hirschmüller: Physiologie und Psychoanalyze im Leben und Werk Josef Breuers. Jahrbuch der Psychoanalyze, Beiheft Nr. 4. Verlag Hans Huber, Bern 1978). ISBN 3456806094: Lower row left: Alexander Crom-Brown, Scottish chemist (date 1923; Journal of the Chemical Society, Transactions, 1923, 123:3242–3,423): Right: Hermann von Helmholtz, German physician, physiologist and physicist (Hermann von Helmholtz, practical Physics, published 1914, Macmillan and Company).

In evolutionary terms, the vestibular system is one of the most ancient senses, however, discovered as one of the latest senses. Its roots date back to the graviceptive statocysts of various invertebrate phyla such as coelenterates. The organ of the statocysts is a fluid-filled cavity with its wall covered by sensory cells which detect the touch of a heavier object floating by the gravitational force to the undermost part of the cyst (20). In vertebrates, the bilateral vestibular labyrinths within the inner ears contain species-specific otolith organs and semicircular canals (21). First experimental concepts for understanding the physiology and function of the vestibular system and its disorders date back to the early 19th century (22). Despite impressive anatomical preparations of the labyrinths, the understanding prevailed that body accelerations were sensed by the motion-induced changes in blood distribution or by skin pressure receptors (23). The foundations of modern vestibular and ocular motor research were laid by a group of 19th century scientists including Purkyne, Mach, Breuer, Helmholtz, and Crum-Brown (24) (Figure 3).

Jan Evangelista Purkynӗ (1787–1869), who founded the doctrine of “Exact Subjectivism*”* in his psychophysical experiments with important contributions to the physiology of vision, (Han, Waddington 2016) the ocular motor, and the vestibular system, nevertheless believed that direct mechanical effects on the cerebellum were responsible for the mechanism of vertigo (25). In 1875, Ernst Mach (1838–1916) published the famous book “Fundamentals of the Theory of Motion Perception” (Grundlinien der Lehre von den Bewegungsempfindungen) (23, 26). Using a rotary chair, Ernst Mach, Josef Breuer (1842–1925), and Alexander Crum-Brown (1838–1922) all suggested that the parameter that is sensed during rotation is angular acceleration in the labyrinth. Contrary to the endolymph flow theory, they presented evidence that the stimulus is a pressure difference across the cupula acting during acceleration (26). Alexander Crum-Brown in his pioneering paper “On the sense of rotation and the anatomy and physiology of the semicircular canals of the internal ear” came to conclusions which are still valid (26). At that time, the central cerebral organization of the vestibular system and its disorders had not yet been investigated and understood (22).

Structural and functional imaging techniques with PET and MRT disclosed a widely distributed bilateral central vestibular network extending from the vestibular nuclei in the caudal brainstem via the thalamus to multiple cortical areas with a dominance in the non-dominant thalamo-cortical hemisphere (27). This made it possible to distinguish not only peripheral from central vestibular disorders but also to attribute central vestibular syndromes topographically to circumscribed lesions within this network (28–30).

### 2.4 Proprioception and pain, senses or just collective terms for specific functions mediated by a multisensory ensemble?

**Figure 4:**
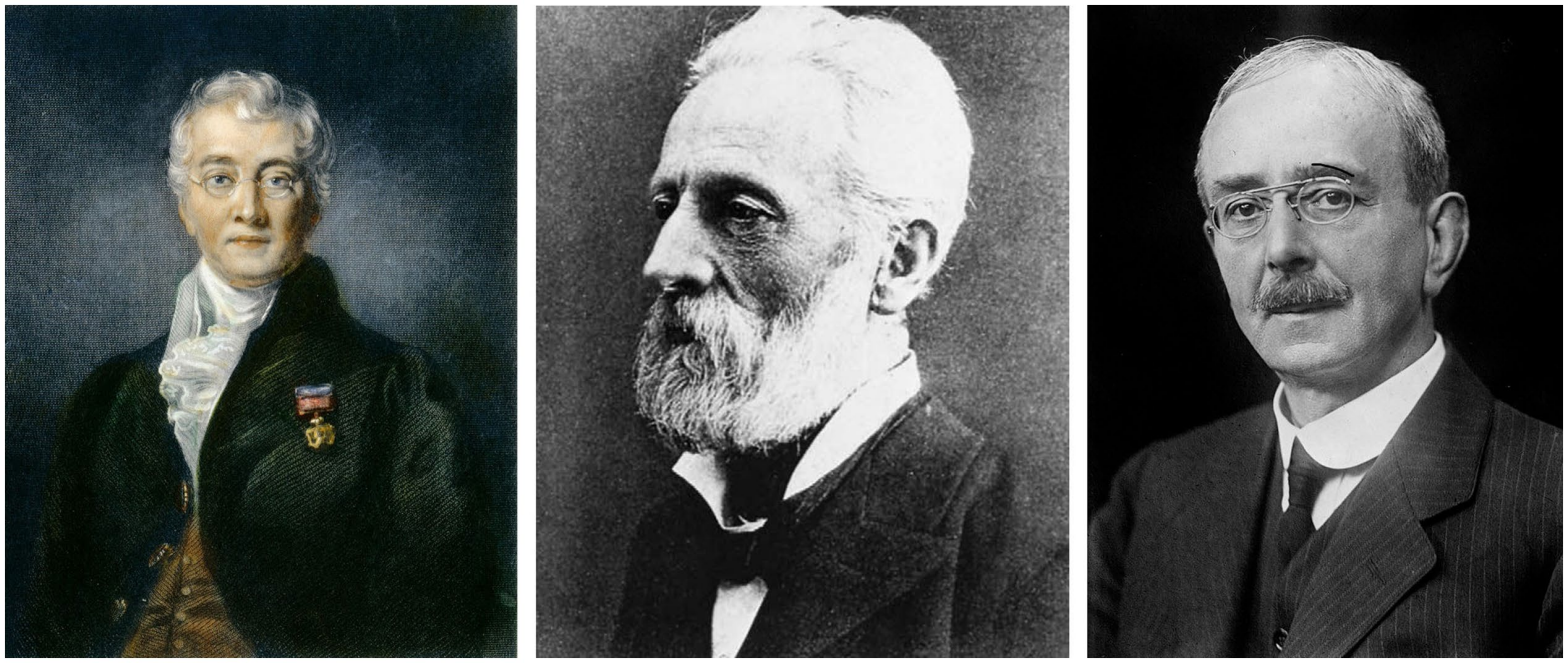
Left: Charles Bell, Scottish surgeon and anatomist (date 1839; http://fineartamerica.com/featured/charles-bell-1774-1842-granger.html). Middle: Henry Charlton Bastian, English neurologist (date: ca. 1900; reprinted in: Jellinek EH. Dr. H C Bastian, scientific Jekyll and Hyde). The Lancet 356 (9248), ss. 2180–2,183 (2001). Right: Charles Scott Sherrington, British neurophysiologist (unknown date; available from the United States Congress’s Prints and Photographs division; https://www.loc.gov/rr/print under the digital ID ggbain.35528).

Before the experimental discovery of a sixth sense—the vestibular system—, the Scottish physiologist Charles Bell (1774–1842) in 1826 described a reciprocal sensorimotor loop between the brain and the muscles which he called a new muscular sense: “Between the brain and the muscles there is a circle of nerves; one nerve conveys the influence from the brain to the muscle, another gives the sense of the condition of the muscle to the brain” (31). Later the English neurologist Henry Charlton Bastian (1837–1915) supported the view that a cortical muscular sense is required for motor coordination of body movements which he termed “kinaesthesia” (32). It was the English neurophysiologist Charles Scott Sherrington (1857–1952) who replaced the term “kinaesthesia” with “proprioception” derived from the Latin words proprius (one’s own) and perceptio (perception). His short definition was: “the perception of joint and body movement as well as position of the body, or body segments, in space” (33). This means that the term included body position and—most importantly—movement of body segments in space and relative to each other [for review, see (2); Figure 4]. Sherrington’s focus on the muscle afferences provided by muscle spindles and Golgi tendon organs—neglecting other senses such as the visual and vestibular system—is understandable because his major scientific interest was spinal reflexes and the first description of the synapse between two connected neurons for which he was awarded the Nobel Prize in Medicine in 1932.

We think that “proprioception” could also meet the criteria of a separate sense, although it often operates in a multisensory mode, preferably together with the visual, vestibular, and somatosensory system. Sensory input from muscle and tendon mechanoreceptors interacts with vestibular and visual motion signals for spatial orientation, gaze, body, limb and balance control. With respect to a different sensory classification schema proprioception is not restricted to interoception (as assumed by some) but connects task-dependent interoception and exteroception. Thus, the often-used term “proprioceptive cervical vertigo” (34) should not be confused with a disorder of simply the cervical muscle sense but refers to the impaired functional achievement of a multisensory ensemble in which somatosensory, vestibular, and visual cues play an important role (35). In other words, structural and functional sensory convergence makes classification difficult.

We hesitate to recommend pain as a sensory entity but regard it as an unpleasant sensation due to stimulation of various unspecific receptors distributed in exteroceptive, interoceptive, and visceroceptive regions. Many pain receptors have multisensory functions such as touch receptors which can also cause pressure dependent pain or skin temperature receptors which cause pain if the stimulus is too hot. Pain may generate helpful topographical hints of certain visceral disorders or warning signals to withdraw the body or body segments in situations which could cause injuries. Chronic pain is an autonomous disease entity (36).

### 2.5 Gustatory sense

A conceptual separation of senses and sensors is much easier, e.g., for the gustatory sensory system, although semantic confusions are not rare in the press and professional literature. As an example, contemporary research has identified two new specific receptors which some in the layman’s and trade press referred to as the discovery of new senses: the four basic tastes of sweet, sour, bitter, and salty were extended first by the Japanese chemist Kikunae Ikeda in 1908 who reported on specific chemoreceptors for glutamates and nucleotides which elicit the pleasant savory taste “umami” (37) and second by the recent description of OTOP1 as a proton-selective ion channel sensor for the taste of ammonium chloride, a combination of bitter, salty, and sour, e.g., known as the confection licorice (38).

### 2.6 Smell—the oldest mammalian sense

From an evolutionary perspective, the chemical sense ‘smell’ is the oldest. Its importance for nutrition, mating, and avoiding environmental danger may be essential for survival. In current medicine, human disorders of smell (anosmia, hyposmia, dysosmia) include neurodegenerative syndromes, such as Parkinson’s or Alzheimer’s diseases, Lewy body dementia as well as immunological disorders like multiple sclerosis, sarcoidosis or head injury and, of particular interest, epidemic Covid-19 disease (39, 40). Disorders of smell and taste can manifest independently—more common in the form of a loss of smell—or as a combination of both (41). Despite the undoubtedly impressive reports on trained dogs tracking odor trails, one should be careful with a generalization and functional explanation especially if it comes to popular statements, e.g., that dogs are 10,000 to 100,000 times more sensitive than humans; the latter is based on an assumed higher number of scent receptors. McGann contradicted the French anatomist Paul Broca who hypothesized that the human evolution of the brain with enlargement of the frontal lobes due to the development of speech, thinking, and the free will led to a relatively smaller and less capable olfactory system. Sigmund Freud, who was very familiar with Broca’s work, supposed that humans are less dependent on instinctive, smell-guided sexual behavior but more driven by civilized rationality (42). Contrary to this historical belief that humans have a poorer sense of smell as compared to other mammalians, it has been argued that humans are even more sensitive to some odors than rodents or dogs and have prominent olfactory bulbs with a similar number of neurons (42). In a thorough review of scientific studies on various mammalian species, the conclusion was that no correlation exists between the olfactory sensitivity, the absolute number of specific olfactory receptors, and their density in the olfactory epithelium or size and quality of the olfactory brain structures (43). For smell and taste, a differentiation between peripheral or central dysfunction is clinically difficult unless imaging clearly shows causative damage. Functions and disorders of the other senses are divided into peripheral and central disorders depending on whether the peripheral organs or the central processing networks are involved.

### 2.7 Higher cortical functions and disorders of the visual or the vestibular system

**Figure 5:**
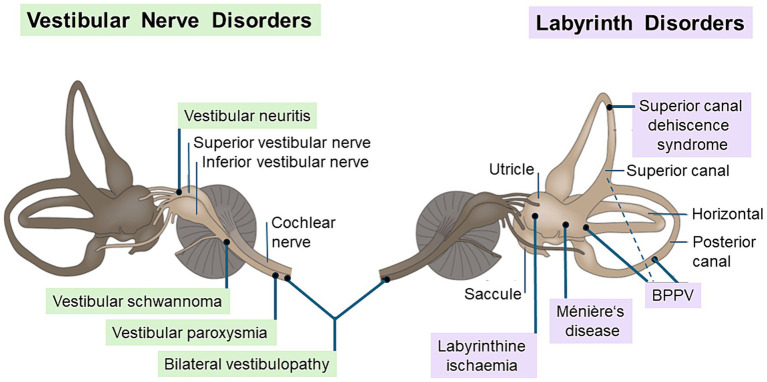
Common peripheral vestibular disorders. The figure shows the causative structure of peripheral vestibular disorders that affect the vestibular nerve (left) or labyrinth (right). Typical vestibular nerve disorders are unilateral vestibulopathy (e.g., vestibular neuritis or vestibular schwannoma), bilateral vestibulopathy, and vestibular paroxysmia due to a neurovascular compression. Labyrintine disorders include benign paroxysmal positional vertigo (BPPV) by canalo- or cupulolithiasis, Menière’s disease with endolymphatic hydrops, superior canal dehiscence syndrome due to a bony defect, and the rare labyrinthine ischemia within the territory of the anterior inferior cerebellar artery [modified from Brandt and Dieterich (28)].

The vestibular system may serve as an example. This is why we selected this system in honor of the late Hans Straka, one of the international leading vestibular neuroscientists with whom the historical review on senses and sensors was initially planned. Vestibular disorders are traditionally classified by the anatomical site of the dysfunction. Lesions of anatomical structures such as the labyrinth and the vestibular nerve, i.e., the first-order neurons, are peripheral (Figure 5).

**Figure 6:**
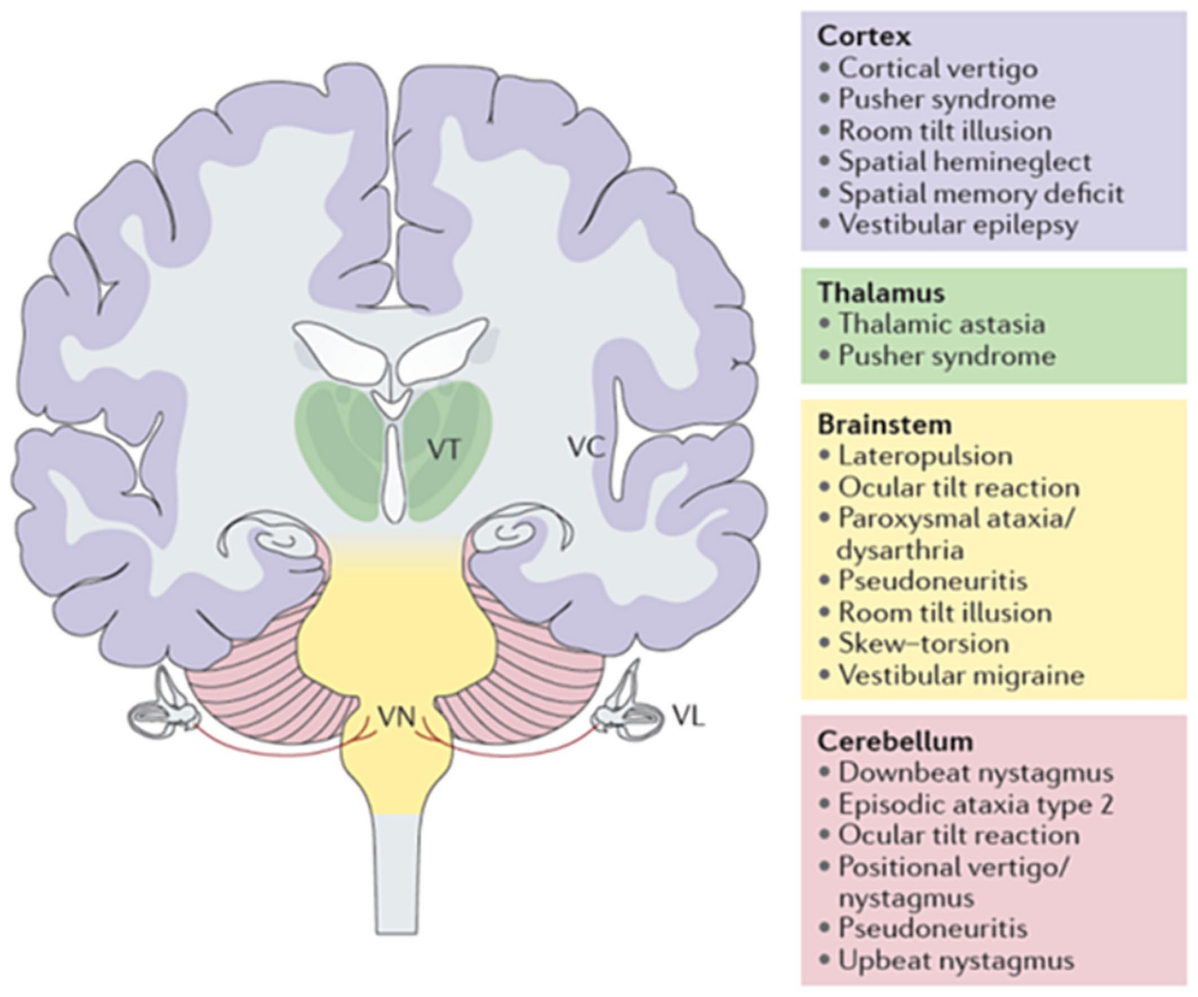
Central vestibular syndromes and disorders of higher vestibular function depending on the lesion site (cortex in purple, thalamus in green, brainstem in yellow, cerebellum in light red). Some disorders have been linked to central lesions at different levels (e.g., the ocular tilt reaction can be caused by brainstem or cerebellar lesions). VC, vestibular cortex; VL, vestibular labyrinth; VN, vestibular nucleus; VT, vestibular thalamus (28).

Lesions of the pontomedullary vestibular nuclei and their pathways from the brainstem to the vestibulo-cerebellum, the thalamus, and cortical vestibular areas are considered part of the bilateral central vestibular network (Figure 6). A simple separation of peripheral from central vestibular disorders disregards a third category, i.e., disorders of *“*higher vestibular” function. In analogy to disorders of *“*higher visual” function, which affect the extrastriate visual cortex, the “what” and “where” pathways, (44–47) a concept of disorders of higher vestibular function was proposed (28, 48, 49). This new category includes the central vestibular system and its interaction with cognition and emotions. Moreover, the hemispheric dominance of the thalamo-cortical vestibular network in various imaging studies (50–56) and meta-analyses (57) is reflected in several neurological disorders involving higher vestibular function. These disorders include impairment of spatial orientation, spatial attention, and balance control, and are based on integration of multimodal interaction. To elucidate the latter, four conditions should be mentioned here: hemispatial neglect, pusher syndrome, the room tilt illusion, and—as an exceptional peripheral condition—bilateral vestibular loss (29). Hemispatial neglect causes an interrupted attention to multimodal sensory stimuli, in particular visual stimuli, within one hemifield contralateral to the acute, mostly right temporo-parietal lesion containing the cortical vestibular network (58, 59). The room tilt illusion is a rare disorder of paroxysmal upside-down vision or of 90° visual tilts which indicates a transient mismatch of the visual and vestibular coordinate systems (28, 60, 61). Pusher syndrome is often under-recognized by neurologists, whereas physical therapists are very familiar with the apparent tilt of the perceived body position in space which the patient attempts to counteract (62–65). A bilateral vestibular loss can also cause deficits of higher vestibular functions associated with hippocampal atrophy. Symptoms include impairment of spatial memory, orientation, and navigation (66–68) which have also been shown in rodents with the Morris-Water-Task (69). Thus, the criterion of higher function is fulfilled if cognition or senses other than the primarily affected ones come into play. *“*The spatial hemineglect and room tilt illusion involve vestibular and visual function to the extent that both conditions can be classified as either disorders of higher vestibular or of higher visual function. A possible way of separating these disorders is to determine whether the causative lesion site affects the vestibular or the visual (48). There are other higher vestibular functions that extend into dimensions of emotion processing such as an overlap of the cerebral anxiety and vestibular networks (70), social cognition (71), or distorted own-body representations (49, 72).

## 3 Conclusion

The three biggest surprises of our selective historical review on the semantic alterations of human sensory systems and sensors from antiquity until the present time were:

1. The five senses—sight, hearing, touch, taste, and smell—listed by Aristotle in his work *“*De Anima” (On the Soul) about 350 BC are still commonly used by non-scientific laypersons after more than 2000 years.
2. The related peripheral sensory organs with their specific sensors—eyes, ears, skin, tongue, and nose—were well known, while the central structures for stimulus processing, perception, and cognition were assumed to reside in the heart rather than the brain in ancient Egypt, Greece, and China.
3. There is an ongoing discussion even among neuroscientists on the number of human sensory systems—six, seven, or eight—and an inconsistent, partly contradictory use of classification features in the literature.

This inconsistent classification is also true for Aristotle’s list. The concept *“*touch” does not describe a sense but a specific function of one of the skin sensors belonging to the “somatosensory system” which contains various sensors for, e.g., pressure, vibration, or temperature. We recommend an update of the classical antique list according to the current state of knowledge with an expansion of the number of senses. This should include the vestibular system for balance control, movement, and gravity perception as, e.g., the sixth sense and proprioception for muscle and tendon receptor-based awareness of the position and movement of the body and parts of the body in space as well as relative to each other as, e.g., the seventh sense. However, pain has been repeatedly assigned to the somatosensory system with a ubiquitous extero- and interoceptive distribution and multiple different stimuli.

Such a comprehensive reorganization requires the cooperation of a group of sensory physiologists, neuroscientists, and experienced physicians involved in the management of patients with sensory and multisensory disorders who are motivated to do the work to improve the current conceptual confusion in this field.

## Author’s note

This article is dedicated to the late biological scientist Hans Straka who was a central and most stimulating character of our common interdisciplinary projects on the vestibular system in animals and humans. Therefore, vestibular function is a major focus of this historical review.

## Author contributions

TB: Writing – original draft, Writing – review & editing. MD: Writing – original draft, Writing – review & editing. DH: Conceptualization, Investigation, Writing – original draft, Writing – review & editing.

## References

[1] Neuhaeuser J. Aristoteles’ Lehre von dem sinnlichen Erkenntnissvermögen und seinen Organen. Leipzig, Verlag von Erich Koschny (L. Heimann’s Verlag). (1878).

[2] Han J Waddington G Adams R Anson J Liu Y. Assessing proprioception: a critical review of methods. J Sport Health Sci. (2016) 5:80–90. doi: 10.1016/j.jshs.2014.10.004, PMID: 30356896 PMC6191985

[3] Freudig D. Lexikon der Biologie. Heidelberg: Spektrum Akademischer Verlag (2006).

[4] Mansfeld J. (2019). Echoes of Theophrastus ’De sensibus’ in Books 4 and 1 of the Aëtian Placita. Rhizomata. 7: 146–167.

[5] Böhme H. Sinne und Blick, Zur mythopoetischen Konstitution des Subjekts. In: Natur und Subjekt, Böhme H (ed). Frankfurt a.M., Suhrkamp. (2008).

[6] Wade NJ Brozek J Hoskovec J. Purkinje’s vision, the dawning of neuroscience New York: Psychology Press (2001).

[7] Paulsen T Rehn R. Timaios. Griechisch/Deutsch. Übersetzung, Anmerkungen und Nachwort von Th. Paulsen und R. Rehn, Stuttgart. (2003).

[8] Aristoteles. Über die. Seele. ed. Dietzingen, Reclam: G. Krapinger (2011).

[9] Wiersma W. Die aristotelische Lehre vom Pneuma. Mnemosyne. (1943) 11:102–7.

[10] Kabelmann. Die Ethnologie der Sinne. https://www.kabelmann.de/content/hausarbeit3_3_1.htm; Ethnologie der Sinne; last accessed 14-03-2024.

[11] Wiltse LL Pait TG. Herophilus of Alexandria (325-255 B. C.): the father of anatomy. Spine. (1998) 23:1904–14. doi: 10.1097/00007632-199809010-00022, PMID: 9762750

[12] Brandt T Huppert D. Brain beats heart: a cross-cultural reflection. Brain. (2021) 144:1617–20. doi: 10.1093/brain/awab080, PMID: 33704411 PMC8320262

[13] Clarke E O'Malley CD. The human brain and spinal cord: A historical study illustrated by writings from antiquity to the twentieth century. San Francisco: Norman Publishing (1996).

[14] Brunner H, Otto, E. Das ägyptische Mundöffnungsritual. I. u. II. Orientalistische Literaturzeitung. (1963) 58:243.

[15] Altenmüller H. Totenliturgie und Mundöffnungsritual. Bemerkungen zur vermuteten «Vision von der Statue im Stein». In: Honi soit qui mal y pense. Studien zum pharaonischen, griechisch-römischen und spätantiken Ehren von Heinz-Josef Thissen. Knuf H, Leitz C, von Recklinghausen D (eds). Leuven, Orientalia Lovanensia Analecta. (2010).

[16] Hippocrates. De morbo sacro De morbo sacro. In: Hippokrates, ausgewählte Schriften. Herausgegeben und übersetzt von Schubert C, Leschhorn W. Düsseldorf/Zürich, Artemis&Winkler (2001).

[17] Elsner N Lüer G. Das Gehirn und sein Geist. Göttingen: Wallstein Verlag (2000).

[18] Brandt T Bauer M Benson J Huppert D. Motion sickness in ancient China: seasickness and cart-sickness. Neurology. (2016) 87:331–5. doi: 10.1212/WNL.0000000000002871, PMID: 27432177

[19] Kovacs J Unschuld P. Essential subtleties on the silver sea - the yin-hai Jing-wei: A Chinese classic on ophthalmology. Berkeley/Los Angeles: University of California Press (1998).

[20] Schmitz J. Graviception in invertebrates In: The senses. Second Ed. Fritzsch, B. Oxford: Elsevier (2020). 88–107.

[21] Platt C Straka H. Vestibular endorgans in vertebrates and adequate sensory stimuli In: The senses. Second Ed. Straka, H. Oxford: Elsevier (2020). 108–28.

[22] Brandt T. Man in motion. Historical and clinical aspects of vestibular function. A review. Brain. (1991) 114:2159–74. doi: 10.1093/brain/114.5.2159 1933240

[23] Henn V Young LR. Ernst Mach on the vestibular organ 100 years ago. ORL J Otorhinolaryngol Relat Spec. (1975) 37:138–48. doi: 10.1159/000275218, PMID: 1093083

[24] Cohen B. Erasmus Darwin's observations on rotation and vertigo. Hum Neurobiol. (1984) 3:121–8. PMID: 6384154

[25] Grusser OJ. J.E. Purkyne's contributions to the physiology of the visual, the vestibular and the oculomotor systems. Hum Neurobiol. (1984) 3:129–44. PMID: 6384155

[26] Henn V. E. Mach on the analysis of motion sensation. Hum Neurobiol. (1984) 3:145–8. PMID: 6384156

[27] Dieterich M Brandt T. Structural and functional imaging of the human bilateral vestibular network from the brainstem to the cortical hemispheres In: The senses. Second ed. Oxford: Elsevier (2020). 414–31.

[28] Brandt T Dieterich M. The dizzy patient: don't forget disorders of the central vestibular system. Nat Rev Neurol. (2017) 13:352–62. doi: 10.1038/nrneurol.2017.58, PMID: 28429801

[29] Brandt T Dieterich M. Central and higher cortical vestibular disorders In: The senses. Second ed. Oxford: Elsevier (2020). 55–68.

[30] Conrad J Baier B Eberle L Ruehl RM Boegle R Zwergal A. Network architecture of verticality processing in the human thalamus. Ann Neurol. (2023) 94:133–45. doi: 10.1002/ana.26652, PMID: 36966483

[31] Bell C. On the nervous circle which connects the voluntary muscles with the brain. Lond Med Phys J. (1826) 1:44–50. PMID: 30494965 PMC5660367

[32] Bastian HC. The “muscular sense”; its nature and cortical location. Brain. (1887) 10:1–89. doi: 10.1093/brain/10.1.1

[33] Sherrington CS. The integrative action of the nervous system. London: Yale University Press (1906).

[34] Gill-Lussier J Saliba I Barthelemy D. Proprioceptive Cervicogenic dizziness care trajectories in patient subpopulations: a scoping review. J Clin Med. (2023) 12:1–22. doi: 10.3390/jcm12051884, PMID: 36902670 PMC10003866

[35] Huppert D. Tsai T. Richter S. (2024). Impact of proprioceptive cervical dizziness in chronic neck pain syndromes on gait and stance during active head-turn challenges. J Neurol, in press. 10.1007/s00415-024-12711-8 PMC11588840 39404783

[36] Raffaeli W Arnaudo E. Pain as a disease: an overview. J Pain Res. (2017) 10:2003–8. doi: 10.2147/JPR.S138864, PMID: 28860855 PMC5573040

[37] Lindemann B Ogiwara Y Ninomiya Y. The discovery of umami. Chem Senses. (2002) 27:843–4. PMID: 12438211 10.1093/chemse/27.9.843

[38] Liang Z Wilson CE Teng B Kinnamon SC Liman ER. The proton channel OTOP1 is a sensor for the taste of ammonium chloride. Nat Commun. (2023) 14:6194. doi: 10.1038/s41467-023-41637-4, PMID: 37798269 PMC10556057

[39] Karamali K Elliott M Hopkins C. COVID-19 related olfactory dysfunction. Curr Opin Otolaryngol Head Neck Surg. (2022) 30:19–25. doi: 10.1097/MOO.0000000000000783 34889850 PMC8711304

[40] Kay LM. COVID-19 and olfactory dysfunction: a looming wave of dementia? J Neurophysiol. (2022) 128:436–44. doi: 10.1152/jn.00255.2022, PMID: 35894511 PMC9377782

[41] Doty RL. Treatments for smell and taste disorders: a critical review. Handb Clin Neurol. (2019) 164:455–79. doi: 10.1016/B978-0-444-63855-7.00025-3, PMID: 31604562

[42] Mc Gann JP. Poor human olfaction is a 19th-century myth. Science. (2017) 356:1–6. 10.1126/science.aam7263 PMC5512720 28495701

[43] Wackermannova M Pinc L Jebavy L. Olfactory sensitivity in mammalian species. Physiol Res. (2016) 65:369–90. PMID: 27070753 10.33549/physiolres.932955

[44] Barton JJ. Disorders of higher visual processing. Handb Clin Neurol. (2011) 102:223–61. doi: 10.1016/B978-0-444-52903-9.00015-7 21601069

[45] De Haan EH Cowey A. On the usefulness of 'what' and 'where' pathways in vision. Trends Cogn Sci. (2011) 15:460–6. doi: 10.1016/j.tics.2011.08.005 21906989

[46] Pelak VS. The clinical approach to the identification of higher-order visual dysfunction in neurodegenerative disease. Curr Neurol Neurosci Rep. (2022) 22:229–42. doi: 10.1007/s11910-022-01186-7, PMID: 35320467

[47] Prasad S Dinkin M. Higher cortical visual disorders. Continuum (Minneap Minn). (2019) 25:1329–61. doi: 10.1212/CON.0000000000000774, PMID: 31584540

[48] Brandt T Strupp M Dieterich M. Towards a concept of disorders of "higher vestibular function". Front Integr Neurosci. (2014) 8:47. 24917796 10.3389/fnint.2014.00047 PMC4041089

[49] Lopez C Nakul E Preuss N Elzière M Mast FW. Distorted own-body representations in patients with dizziness and during caloric vestibular stimulation. J Neurol. (2018) 265:86–94. doi: 10.1007/s00415-018-8906-8, PMID: 29876763

[50] Becker-Bense S Willoch F Stephan T Brendel M Yakushev I Habs M. Direct comparison of activation maps during galvanic vestibular stimulation: a hybrid H2[15 O] PET-BOLD MRI activation study. PLoS One. (2020) 15:e0233262. doi: 10.1371/journal.pone.0233262, PMID: 32413079 PMC7228124

[51] Dieterich M Bense S Lutz S Drzezga A Stephan T Bartenstein P. Dominance for vestibular cortical function in the non-dominant hemisphere. Cereb Cortex. (2003) 13:994–1007. doi: 10.1093/cercor/13.9.994, PMID: 12902399

[52] Dieterich M Kirsch V Brandt T. Right-sided dominance of the bilateral vestibular system in the upper brainstem and thalamus. J Neurol. (2017) 264:55–62. doi: 10.1007/s00415-017-8453-8 28315957

[53] Ertl M Klaus M Mast FW Brandt T Dieterich M. Spectral fingerprints of correct vestibular discrimination of the intensity of body accelerations. NeuroImage. (2020) 219:117015. doi: 10.1016/j.neuroimage.2020.117015, PMID: 32505699

[54] Ertl M Moser M Boegle R Conrad J zu Eulenburg P Dieterich M. The cortical spatiotemporal correlate of otolith stimulation: vestibular evoked potentials by body translations. NeuroImage. (2017) 155:50–9. doi: 10.1016/j.neuroimage.2017.02.044, PMID: 28254458

[55] Kirsch V Boegle R Keeser D Kierig E Ertl-Wagner B Brandt T. Handedness-dependent functional organizational patterns within the bilateral vestibular cortical network revealed by fMRI connectivity based parcellation. NeuroImage. (2018) 178:224–37. doi: 10.1016/j.neuroimage.2018.05.018, PMID: 29787866

[56] MC Assey M Brandt T Dieterich M. EEG analysis of the visual motion activated vection network in left- and right-handers. Sci Rep. (2022) 12:19566. 36379961 10.1038/s41598-022-21824-x PMC9666650

[57] Zu EP Caspers S Roski C. Meta-analytical definition and functional connectivity of the human vestibular cortex. NeuroImage. (2012) 60:162–9. 22209784 10.1016/j.neuroimage.2011.12.032

[58] Karnath HO Dieterich M. Spatial neglect--a vestibular disorder? Brain. (2006) 129:293–305. doi: 10.1093/brain/awh698, PMID: 16371409

[59] Vallar G Perani D. The anatomy of unilateral neglect after right-hemisphere stroke lesions. A clinical/CT-scan correlation study in man. Neuropsychologia. (1986) 24:609–22. doi: 10.1016/0028-3932(86)90001-1, PMID: 3785649

[60] Brandt T. Cortical matching of visual and vestibular 3D coordinate maps. Ann Neurol. (1997) 42:983–4. doi: 10.1002/ana.410420624, PMID: 9403495

[61] Sierra-Hidalgo F de Pablo-Fernández E Herrero-San Martín A Correas-Callero E Herreros-Rodríguez J Romero-Muñoz JP. Clinical and imaging features of the room tilt illusion. J Neurol. (2012) 259:2555–64. doi: 10.1007/s00415-012-6536-0, PMID: 22588254

[62] Baier B Janzen J Müller-Forell W Fechir M Müller N Dieterich M. Pusher syndrome: its cortical correlate. J Neurol. (2012) 259:277–83. doi: 10.1007/s00415-011-6173-z, PMID: 21830093

[63] Karnath HO. Pusher syndrome--a frequent but little-known disturbance of body orientation perception. J Neurol. (2007) 254:415–24. doi: 10.1007/s00415-006-0341-6, PMID: 17385082

[64] Pedersen PM Wandel A Jørgensen HS Nakayama H Raaschou H Olsen T. Ipsilateral pushing in stroke: incidence, relation to neuropsychological symptoms, and impact on rehabilitation. The Copenhagen stroke study. Arch Phys Med Rehabil. (1996) 77:25–8. doi: 10.1016/S0003-9993(96)90215-4, PMID: 8554469

[65] Perennou DA Mazibrada G Chauvineau V Greenwood R Rothwell J Gresty MA. Lateropulsion, pushing and verticality perception in hemisphere stroke: a causal relationship? Brain. (2008) 131:2401–13. doi: 10.1093/brain/awn170, PMID: 18678565

[66] Brandt T Schautzer F Hamilton DA Brüning R Markowitsch HJ Kalla R. Vestibular loss causes hippocampal atrophy and impaired spatial memory in humans. Brain. (2005) 128:2732–41. doi: 10.1093/brain/awh617, PMID: 16141283

[67] Kremmyda O Hufner K Flanagin VL. Beyond dizziness: virtual navigation, spatial anxiety and hippocampal volume in bilateral vestibulopathy. Front Hum Neurosci. (2016) 10:139. 27065838 10.3389/fnhum.2016.00139 PMC4814552

[68] Previc FH Krueger WW Ross RA. The relationship between vestibular function and topographical memory in older adults. Front Integr Neurosci. (2014) 8:46. 24917795 10.3389/fnint.2014.00046 PMC4041072

[69] Smith PF Zheng Y. From ear to uncertainty: vestibular contributions to cognitive function. Front Integr Neurosci. (2013) 7:84. 24324413 10.3389/fnint.2013.00084 PMC3840327

[70] Neumann N Fullana MA Radua J Brandt T Dieterich M Lotze M. Common neural correlates of vestibular stimulation and fear learning: an fMRI meta-analysis J Neurol. (2003) 270:1843–1856. doi: 10.1007/s00415-023-11568-7, PMID: 36723684 PMC10025232

[71] Lopez C. The vestibular system: balancing more than just the body. Curr Opin Neurol. (2016) 29:74–83. doi: 10.1097/WCO.0000000000000286 26679566

[72] Lopez C Elziere M. Out-of-body experience in vestibular disorders - a prospective study of 210 patients with dizziness. Cortex. (2018) 104:193–206. doi: 10.1016/j.cortex.2017.05.026, PMID: 28669509

